# Efficacy of elotuzumab for multiple myeloma in reference to lymphocyte counts and kappa/lambda ratio or B2 microglobulin

**DOI:** 10.1038/s41598-023-32426-6

**Published:** 2023-03-29

**Authors:** Yutaka Shimazu, Junya Kanda, Satoru Kosugi, Tomoki Ito, Hitomi Kaneko, Kazunori Imada, Yuji Shimura, Shin-ichi Fuchida, Kentaro Fukushima, Hirokazu Tanaka, Satoshi Yoshihara, Kensuke Ohta, Nobuhiko Uoshima, Hideo Yagi, Hirohiko Shibayama, Ryosuke Yamamura, Yasuhiro Tanaka, Hitoji Uchiyama, Yoshiyuki Onda, Yoko Adachi, Hitoshi Hanamoto, Ryoichi Takahashi, Mitsuhiro Matsuda, Takashi Miyoshi, Teruhito Takakuwa, Masayuki Hino, Naoki Hosen, Shosaku Nomura, Chihiro Shimazaki, Itaru Matsumura, Akifumi Takaori-Kondo, Junya Kuroda

**Affiliations:** 1 Department of Hematology and Oncology Graduate School of Medicine, Kyoto University, 54, Kawaramachi, Shogoin, Sakyoku, Kyoto, 606-8507 Japan; 2 Department of Internal Medicine (Hematology), Toyonaka Municipal Hospital, Toyonaka, Japan; 3 First Department of Internal Medicine, Kansai Medical University, Hirakata, Japan; 4 Department of Hematology, Japanese Red Cross Osaka Hospital, Osaka, Japan; 5 Division of Hematology and Oncology, Department of Medicine, Kyoto Prefectural University of Medicine, Kyoto, Japan; 6 Department of Hematology, Japan Community Health Care Organization Kyoto Kuramaguchi Medical Center, Kyoto, Japan; 7 Department of Hematology and Oncology, Osaka University Graduate School of Medicine, Suita, Japan; 8 Department of Hematology and Rheumatology, Kindai University Faculty of Medicine, Higashiōsaka, Japan; 9 Division of Hematology, Department of Internal Medicine, Hyogo College of Medicine, Nishinomiya, Japan; 10 Hematology Ohta Clinic, Shinsaibashi, Japan; 11 Department of Hematology, Japanese Red Cross Kyoto Daini Hospital, Kyoto, Japan; 12 Department of Hematology and Oncology, Nara Prefecture General Medical Center, Nara, Japan; 13 Department of Hematology, National Hospital Organization Osaka National Hospital, Osaka, Japan; 14 Department of Hematology, Osaka Saiseikai Nakatsu Hospital, Osaka, Japan; 15 Department of Hematology, Japanese Red Cross Wakayama Medical Center, Wakayama, Japan; 16 Department of Hematology, Japanese Red Cross Kyoto Daiichi Hospital, Kyoto, Japan; 17 Department of Hematology, Japanese Red Cross Takatsuki Hospital, Takatsuki, Japan; 18 Department of Internal Medicine, Japan Community Health Care Organization Kobe Central Hospital, Kobe, Japan; 19 Department of Hematology, Kindai University Nara Hospital, Ikoma, Japan; 20 Department of Hematology, Omihachiman Community Medical Center, Omihachiman, Japan; 21 Department of Hematology, PL General Hospital, Tondabayashi, Japan; 22 Department of Hematology, Uji Tokushukai Hospital, Uji, Japan; 23 Department of Hematology, Osaka Metropolitan University Graduate School of Medicine, Osaka, Japan

**Keywords:** Immunotherapy, Lymphocytes, Tumour immunology, Translational research, Predictive markers

## Abstract

Novel therapeutic drugs have dramatically improved the overall survival of patients with multiple myeloma. We sought to identify the characteristics of patients likely to exhibit a durable response to one such drug, elotuzumab, by analyzing a real-world database in Japan. We analyzed 179 patients who underwent 201 elotuzumab treatments. The median time to next treatment (TTNT) with the 95% confidence interval was 6.29 months (5.18–9.20) in this cohort. Univariate analysis showed that patients with any of the following had longer TTNT: no high risk cytogenic abnormalities, more white blood cells, more lymphocytes, non-deviated κ/λ ratio, lower β_2_ microglobulin levels (B2MG), fewer prior drug regimens, no prior daratumumab use and better response after elotuzumab treatment. A multivariate analysis showed that TTNT was longer in patients with more lymphocytes (≥ 1400/μL), non-deviated κ/λ ratio (0.1–10), lower B2MG (< 5.5 mg/L) and no prior daratumumab use. We proposed a simple scoring system to predict the durability of the elotuzumab treatment effect by classifying the patients into three categories based on their lymphocyte counts (0 points for ≥ 1400/μL and 1 point for < 1400/μL) and κ/λ ratio (0 points for 0.1–10 and 1 point for < 0.1 or ≥ 10) or B2MG (0 points for < 5.5 mg/L and 1 point for ≥ 5.5 mg/L). The patients with a score of 0 showed significantly longer TTNT (*p* < 0.001) and better survival (*p* < 0.001) compared to those with a score of 1 or 2. Prospective cohort studies of elotuzumab treatment may be needed to validate the usefulness of our new scoring system.

## Introduction

The introduction of new drugs, particularly the monoclonal antibodies (mAbs) elotuzumab, daratumumab, and isatuximab, has dramatically improved the prognosis of patients with multiple myeloma (MM)^1–4^. Patients with relapsed MM currently receive dexamethasone, proteosome inhibitors, immunomodulatory drugs, mAbs or a combination of these drugs. Among the mAbs, the anti-CD38 antibodies daratumumab^1^ and isatuximab^3^ are widely used for relapsed MM patients and are associated with a high response rate and superior prognosis. There is another mAb called elotuzumab, which is an antibody against signaling lymphocytic activation molecule F7 (SLAMF7)^5^. Elotuzumab is used with the combination of lenalidomide or pomalidomide in a relapsed setting^2,5,6^. Elotuzumab is effective against SLAMF7-expressing myeloma via active immune cells, particularly natural killer cells^7,8^. Although previous studies reported that elotuzumab showed a high response rate in relapsed MM patients, durable efficacy was achieved in only one fourth of the patients^2,6^. In addition, there are no appropriate biomarkers to predict the durable efficacy of elotuzumab before administration. Because non-responders might benefit from alternative treatments, it is important to select suitable patients for elotuzumab treatment beforehand.

In this study, we attempted to identify biomarkers of the suitability of patients for elotuzumab treatment by focusing on the immunological mechanism of elotuzumab. Elotuzumab is not a cytotoxic drug but rather an immunotherapeutic that works with the help of host immune cells. Therefore, it is important to take into account the host’s immune status. Here, we hypothesized that the balance between the tumor burden and the immune status of the host before treatment may determine the efficacy of elotuzumab treatment. To examine this hypothesis, we conducted a retrospective observational analysis using the real-world database collected by the Kansai Myeloma Forum (KMF) in Japan.

## Patients and methods

### Data source and patients

The KMF is a study group consisting of 123 physicians from 46 facilities in Japan. The KMF database includes the physician-reviewed real-world clinical data of patients with plasma cell dyscrasias and their periodic follow-ups. This study was approved by the Data Management Committee of the Graduate School of Medicine, Kyoto University, and by the institutional review board (approval no. R2887).

A total of 4,095 patients with plasma cell dyscrasias were registered in the KMF database as of February 2021. All the patients were diagnosed as having MM or MM-related disorders based on institutional assessment. From the KMF database, we selected patients who fulfilled the following inclusion criteria: symptomatic MM, age over 20, treatment for relapsed or refractory MM, and treatment with elotuzumab between November 2016 and February 2021 (after its approval for clinical use). Because elotuzumab was approved only for the treatment of relapsed or refractory MM in Japan, elotuzumab was used as a second-or later-line treatment in all cases. A total of 201 patients met the above inclusion criteria for this study. We conducted secondary research to collect the laboratory data 1–7 days before cycle 1 day 1 elotuzumab treatment (after the previous treatment). After excluding 22 patients for lack of data, we finally analyzed 179 relapsed MM patients who underwent a total of 201 treatments with elotuzumab.

The patient responses to treatment were assessed based on the criteria of the international uniform response criteria^9^ for multiple myeloma. The best responses against elotuzumab were classified by institutional physicians into five categories: complete response (CR), very good partial response (VGPR), partial response (PR), stable disease (SD), and progressive disease (PD).

We included the data related to high risk cytogenetic abnormalities based on the physicians' input data by referring to the consensus of the International Myeloma Working group^10^, which includes the deletions 17p, t(4;14) and t(14;16). Unfavorable cytogenetic abnormalities were categorized by a fluorescence in situ hybridization analysis.

### Statistical analyses

The histogram of white blood cell (WBC) counts, neutrophil counts, monocyte counts, lymphocyte counts, β_2_ microglobulin (B2MG) and κ/λ ratio were shown in Fig. S1. To determine the cutoff value, we applied the cutoff value of β_2_ microglobulin (B2MG) according to the International Staging system for MM^11^ as it has been widely accepted (Fig. S2A). As there were no fixed cut off values for other laboratory parameters, we tested the 25th, 50th and 75th percentile value as potential cut-off values (Fig. S2B–E).We referred to prior studies analyzing the overall survival (OS) of immune therapies which reported lymphocyte counts of 500–1500/μL as the threshold^12–15^. We also took into account that only one fourth of the patients were able to obtain durable efficacy by elotuzumab treatment^2,6^ and determined the cut off values for WBC counts, neutrophile counts, lymphocyte counts, monocyte counts and κ/λ ratio as 3500/μl, 3000/μl, 1400/μl, 300/μl and 0.1–10, respectively.

We chose the time to the next treatment (TTNT) as primary endpoint instead of progression free survival (PFS) for our retrospective analysis^16,17^, since the timing of progressive disease is difficult to precisely determine in our cohort. We included the TTNT for elotuzumab treatment, which was calculated from the time of elotuzumab treatment until the date of next treatment, death by any cause or the date of last contact. The data were censored for the date of next treatment when the cessation of elotuzumab treatment was planned advance. We calculated TTNT depending on each treatment. To analyze the underlying factors affecting the TTNT of elotuzumab treatment, we selected the following parameters. First, we selected the type of treatment regimen: elotuzumab plus lenalidomide (ERd) regimen and/or elotuzumab plus pomalidomide (EPd) regimen. Next, we adapted two parameters to estimate the tumor burden of myeloma: the κ/λ ratio and B2MG. These two parameters are recognized to reflect tumor burden^11,18,19^. And finally, we adopted several parameters which, based on our clinical experience, appear to be correlated with the host immune status: white blood cell counts and other leukocyte fractions. We selected the leukocyte fractions (neutrophil, lymphocyte, or monocyte counts) which showed significant correlation with TTNT on univariate analysis and used them in the subsequent analysis.

To calculate the OS, only the data for the patients with first elotuzumab treatment were analyzed. The survival curves of TTNT and OS were plotted using the Kaplan–Meier method, and the log-rank test was used for comparisons among groups. The Cox proportional hazard model was used to calculate the hazard ratios (HRs) for each variable along with the 95% confidence interval (CI). Variables considered in the univariate analysis were age, gender, type of treatment regimen, high risk cytogenic abnormalities, WBC counts, neutrophil counts, lymphocyte counts, monocyte counts, κ/λ ratio, B2MG, number of elotuzumab treatments, number of prior regimens and prior use of daratumumab. A multivariate analysis was conducted for all the variables except the high-risk cytogenic abnormalities that showed p values of less than 0.1 in a univariate analysis. Presence of high-risk cytogenetic abnormality was excluded from the multivariate analysis because more than 40% of cases lacked the requisite data. We also adjusted the OS by the significant factors in multivariate analysis.

To establish a predictive model for the durability of elotuzumab treatment, we used two different sets of multivariate analyses. We used two different parameters to estimate the tumor burden of myeloma: the κ/λ ratio and B2MG. In model 1, we adapted the type of treatment regimen, the κ/λ ratio and the lymphocyte counts which showed significant correlation with TTNT on univariate analysis as parameters. In model 2, we adapted the type of treatment regimen, the B2MG and the leukocyte fractions which showed significant correlation with TTNT on univariate analysis as parameters. As we found weak correlation between WBC counts and lymphocyte counts (correlation coefficient: 0.585) and very weak correlation between monocyte counts and WBC counts (correlation coefficient: 0.468) or lymphocyte counts (correlation coefficient: 0.339), we selected lymphocyte counts in our models. We used C statistics (C-index) to evaluate the predictive accuracy of the models^20,21^. The c-index of an ideal test became closer to 1.0. We used the bootstrap method to validate our results of the scoring system^22,23^. In each step, 1000 bootstrap samples with replacements were created from the dataset. All statistical analyses were performed using the EZR (ver. 1.54) software package (Saitama Medical Center/Jichi Medical University, Saitama, Japan)^24^ along with a graphical user interface for the R software package (ver. 4.0.3; The R Foundation for Statistical Computing) or SPSS software (ver. 28; IBM, USA). *P*-values < 0.05 were considered significant in all analyses.

### Ethical approval

All procedures performed in this study involving the patient were in accordance with the ethical standards of Kyoto University Graduate School and Faculty of Medicine, Ethics Committee institutional (approval no. R2887, approved date: January 6th, 2022) and with the 1964 Helsinki declaration and its later amendments or comparable ethical standards. The informed consent requirement for this retrospective study was waived by Kyoto University Graduate School and Faculty of Medicine, Ethics Committee institutional because the study was conducted retrospectively and the opportunity to refuse was guaranteed.

## Results

### TTNT of elotuzumab in relapsed MM

The characteristics of patients are summarized in Table 1. A total of 201 elotuzumab treatments in 179 patients were analyzed. The median age at the elotuzumab treatment was 71 years. The numbers of patients treated with ERd regimen and EPd regimen were 146 (72.6%) and 55 (27.4%), respectively. The median number of prior regimens was 4, and about 90% of cases were treated with immunomodulatory drugs and/or proteasome inhibitors prior to elotuzumab treatment. Daratumumab was used in 53 (26.4%) cases. Elotuzumab was administered for a second time in 22 (10.9%) cases: 16 cases received ERd followed by EPd regimen, 5 cases received ERd followed by ERd regimen and 1 case received EPd followed by EPd regimen. A histogram of the laboratory data is shown in Suppl. Fig. S1. The number of patients showing a response to elotuzumab, which included those with a CR, VGPR or PR, was 83 (41.3%) in this cohort (Fig. S3).

**Table 1:** Characteristics of multiple myeloma patients.

		Elotuzumab treatment
Number of patients		179
Total number of treatments		201
Median age (years) at elotuzumab treatment	Median (range)	71 (20–87)
Gender	Male	100 (55.9%)
	Female	79 (44.1%)
Type of treatment regimen	ERd	146 (72.6%)
	EPd	55 (27.4%)
Type of heavy chain	IgG	115 (64.2%)
	IgA	31 (17.3%)
	IgM	2 (1.1%)
	Not detected	31 (17.3%)
Type of light chain	λ	73 (40.8%)
	κ	105 (58.7%)
	NA	1 (0.6%)
ISS stage at diagnosis	I	53 (29.6%)
	II	64 (35.8%)
	III	48 (26.8%)
	NA	14 (7.8%)
High risk cytogenic abnormality	del (17)	12 (6.7%)
	t(4;14)	12 (6.7%)
	t(14;16)	3 (1.7%)
	None of above abnormalities	78 (43.6%)
	NA	74 (41.3%)
Laboratory data before elotuzumab treatment		
White blood cell count (/μL, median, range)		3600 (900–11,900)
Neutrophil count (/μL, median, range)		2019 (216–8888)
Lymphocyte count (/μL, median, range)		1065 (39–4403)
Monocyte count (/μL, median, range)		284 (13–1420)
Free light chain (mg/L, median, range)	κ	17.8 (0–6010)
	λ	13.6 (0–10,900)
	κ/λ ratio	1.1 (0.001–3320)
B2MG (mg/L, median, range)		2.9 (0.15–30.7)
IgG (mg/dL, median, range)		788 (45–10,500)
IgA (mg/dL, median, range)		42 (1–4813)
IgM (mg/dL, median, range)		14 (1–2286)
Bone marrow infiltration of plasma cell (%)	Median (range)	4.6 (0.0–78.7)
Prior regimen numbers	Median (range)	4 (1–20)
Prior treatments	IMIDs	181 (90.0%)
	PI	184 (91.5%)
	Daratumumab	53 (26.4%)
	Elotuzumab	22 (10.9%)
	Autologous SCT	64 (35.8%)
	Allogenic SCT	3 (1.7%)
Follow-up period of survivors (median days, range)		553 (14–1726)

The characteristics of multiple myeloma patients who were treated with elotuzumab are shown in Table 1. Laboratory data were collected before the elotuzumab treatment.

ERd, EPd, NA, IMIDs, PI and B2MG. ERd: elotuzumab, lenalidomide and dexamethasone regimen; EPd: elotuzumab, pomalidomide and dexamethasone regimen; NA: not available; IMIDs: immunomodulatory drugs; PI: proteosome inhibitor; B2MG: β_2_ microglobulin; SCT: stem cell transplantation.

**Figure 1:**
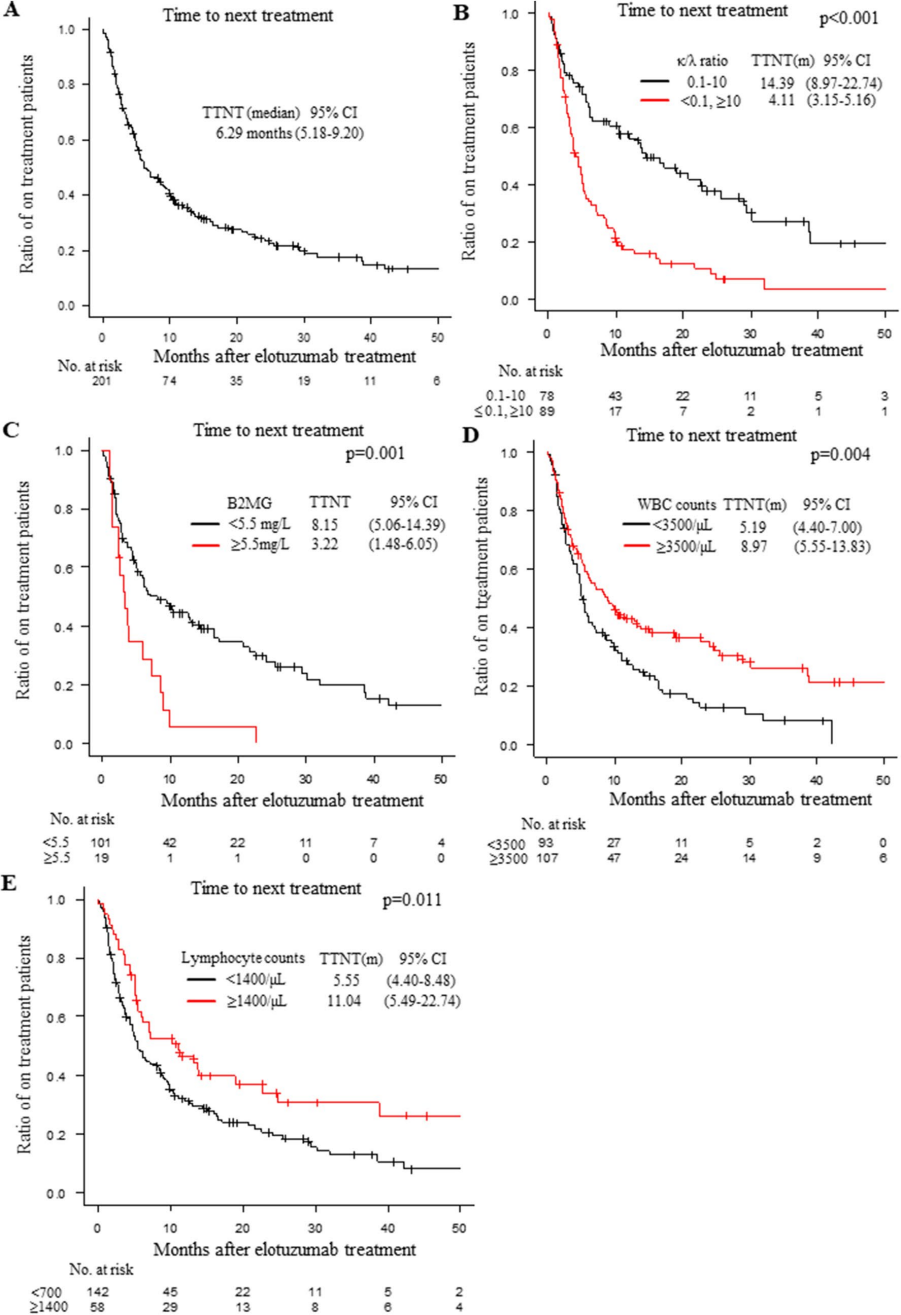
(**A**) The time to next treatment (TTNT) of the multiple myeloma (MM) patients treated with elotuzumab. The median TTNT (months) and 95% CI are shown. (**B**) The TTNT of the MM patients treated with elotuzumab classified according to the κ/λ ratio: 0.1 to 10 (*black*) and less than 0.1 or 10 or more (*red*). The median TTNT (months) and 95% CI are shown. (**C**) The TTNT of the MM patients treated with elotuzumab according to the β_2_ microglobulin (B2MG); less than 5.5 mg/L (*black*) and 5.5 mg/L or more (*red*). The median TTNT (months) and 95% CI are shown. (**D**) The TTNT of the MM patients treated with elotuzumab classified according to the white blood cell (WBC) counts: less than 3500/μL (*black*) and 3500/μL or more (*red*). The median TTNT (months) and 95% CI are shown. (**E**) The TTNT of the MM patients treated with elotuzumab classified according to the lymphocyte counts: less than 1400/μL (*black*) and 1400/μL or more (*red*). The median TTNT (months) and 95% CI are shown. The number of patients at risk in each group is shown in the lower panel of each figure. CI: confidence interval.

TTNT of elotuzumab was 6.29 months (with a 95% CI of 5.18–9.20: Fig. 1A). When we compared the TTNT according to the regimen, the TTNTs of the ERd regimen and EPd regimen were 7.10 (5.49–9.99) and 4.86 (2.99–9.92) months, respectively (Fig. S4A; *p* = 0.076). The TTNTs of the first and second administrations of elotuzumab were 7.00 (5.36–9.89) and 4.86 (1.38–9.93) months, respectively (Fig. S4B, *p* = 0.149).

### The underlying factors affecting the TTNT of elotuzumab

To analyze the underlying factors affecting the TTNT of elotuzumab treatment, we analyzed two parameters: the κ/λ ratio and B2MG. The TTNT of elotuzumab treatment was longer in the patients with a non-deviated κ/λ ratio (κ/λ ratio of 0.1–10) (*p* < 0.001; Fig. 1B and Table 2) and in patients with a lower B2MG (< 5.5 mg/L) (*p* = 0.001; Fig. 1C, Table 2 and Fig. S2B) before elotuzumab treatment. When we analyzed the TTNT according to WBC and lymphocyte counts, the patients with higher WBC counts (≥ 3500/μl) before elotuzumab treatment showed longer TTNT than the patients with lower WBC counts (*p* = 0.004; Fig. 1D and Table 2). The patients with higher lymphocyte counts (≥ 1400/μl) showed longer TTNT (*p* = 0.011, Fig. 1E, Table 2 and Fig. S2A). There was no correlation between TTNT and neutrophil or monocyte counts (Table 2 and Fig. S5A-B).

**Table 2:** Univariate analysis for TTNT.

Factors		Univariate analysis		
		TTNT (months)	95% CI	*p*-value
Age at elotuzumab treatment	< 65 years	7	3.58–9.66	0.191
	≥ 65 years	6.08	5.09–10.28	
Gender	Male	6.21	5.19–9.99	0.954
	Female	6.08	4.40–10.05	
Type of treatment regimen	ERd	7.1	5.49–9.99	0.076
	EPd	4.86	2.99–9.92	
High risk cytogenic abnormalities	None	11.96	7.10–17.08	0.002
	One or more	5.09	2.10–6.51	
White blood cell counts	< 3500/μl	5.19	4.40–7.00	0.004
	≥ 3500/μl	8.97	5.55–13.83	
Neutrophil counts	< 3000/μl	6.05	4.90–8.74	0.102
	≥ 3000/μl	8.64	4.40–22.74	
Lymphocyte counts	< 1400/μl	5.55	4.40–8.48	0.011
	≥ 1400/μl	11.04	5.49–22.74	
Monocyte counts	< 300/μl	6.21	4.90–12.68	0.311
	≥ 300/μL	10.32	6.05–17.08	
κ/λ ratio	0.1–10	14.39	8.97–22.74	< 0.001
	< 0.1, ≥ 10	4.11	3.15–5.16	
B2MG	< 3.5 mg/L	8.15	5.06–14.39	0.001
	≥ 3.5 mg/L	3.22	1.48–6.05	
Number of treatments	First	7	5.36–9.89	0.149
	Second	4.86	1.38–9.92	
Prior regimen numbers	< 4	18.96	7.10-NA	< 0.001
	≥ 4	5.36	4.50–7.13	
Prior use of daratumumab	No	8.97	5.85–13.17	< 0.001
	Yes	3.65	2.30–5.49	

TTNT was calculated from the time of elotuzumab treatment to the time of next treatment. Univariate analyses against TTNT in MM patients treated with elotuzumab were performed for each factor. The log-rank test was used for comparisons among groups. TTNT (months) is shown with the 95% confidence interval (CI) and *p*-value.

TTNT, CI, ERd, EPd, B2MG and NA. TTNT: time to next treatment; CI: confidence interval; ERd: elotuzumab, lenalidomide and dexamethasone regimen; EPd: elotuzumab, pomalidomide and dexamethasone regimen; B2MG: β_2_ microglobulin; NA: not available.

We performed a univariate analysis to clarify other factors correlated with TTNT and found that patients with the following showed better TTNT: no high risk cytogenic abnormalities, fewer than 4 prior regimens, and no prior daratumumab use (Table 2 and Fig. S6A,B). The TTNT of the patients with prior use of daratumumab was shorter (8.97 months vs 3.65 months), and this relation was independent of the period from the last daratumumab administration to the elotuzumab treatment (Fig. S6C). Also, the patients with prior use of daratumumab had lower lymphocyte counts (Fig. S7).

### Prediction model for elotuzumab treatment

Next, we performed a multivariate analysis to determine factors related to the TTNT of elotuzumab; we included all factors that showed p values of less than 0.1 in the univariate analysis. The factors independently associated with superior TTNT were lymphocyte counts ≥ 1400/μL (*p* = 0.009), non-deviated κ/λ ratio (*p* = 0.050), B2MG < 5.5 mg/L (*p* = 0.008) and nonuse of daratumumab before elotuzumab treatment (*p* < 0.001; Table 3).

**Table 3:** Multivariate analysis for TTNT.

Factors		Multivariate analysis		
		Hazard ratio	95% CI	*p*-value
Type of treatment regimen	ERd	1		0.202
	EPd	0.694	0.395–1.217	
White blood cell counts	< 3500/μl	1		0.776
	≥ 3500/μl	0.935	0.591–1.481	
Lymphocyte counts	< 1400/μl	1		0.009
	≥ 1400/μl	0.491	0.289–0.835	
κ/λ ratio	0.1–10	1		0.05
	< 0.1, ≥ 10	1.628	0.999–2.653	
B2MG	< 5.5 mg/L	1		0.008
	≥ 5.5 mg/L	2.09	1.212–3.605	
Prior regimen numbers	< 4	1		0.088
	≥ 4	1.718	0.923–3.198	
Prior use of daratumumab	No	1		< 0.001
	Yes	3.009	1.815–4.989	

Multivariate analyses against TTNT in MM patients treated with elotuzumab were performed using the factors that showed *p* < 0.1 in univariate analysis. The Cox proportional hazard model was used to calculate the hazard ratio for each variable; the 95% CI and *p*-value are shown.

TTNT, CI, ERd, EPd and B2MG. TTNT: time to next treatment; CI: confidence interval; ERd: elotuzumab, lenalidomide and dexamethasone regimen; EPd: elotuzumab, pomalidomide and dexamethasone regimen; B2MG: β_2_ microglobulin.

**Figure 2:**
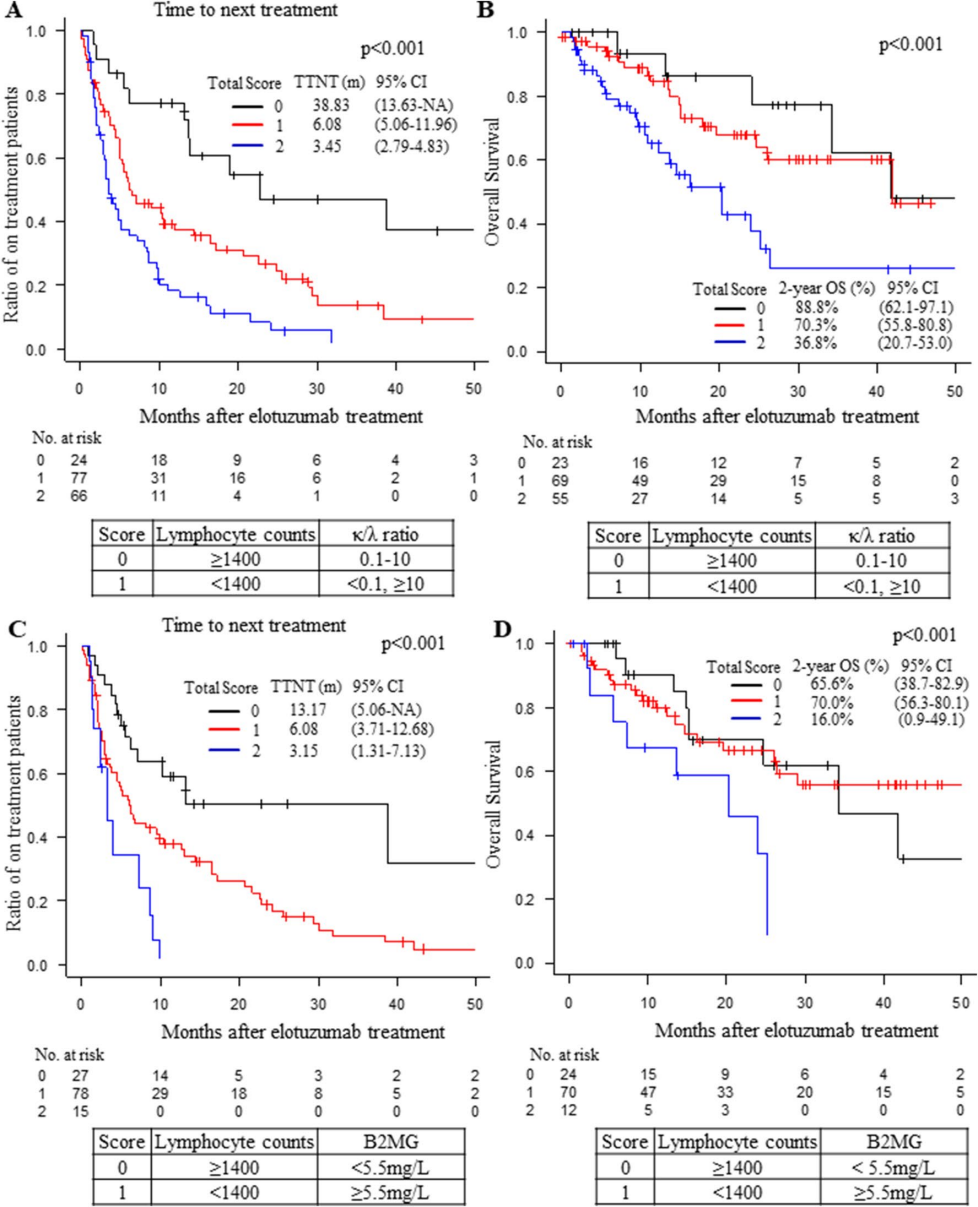
(**A**) The TTNT of the MM patients treated with elotuzumab according to a three-point scoring system: 0 points (*black*), 1 point (*red*) and 2 points (*blue*). Total scores were calculated according to the lymphocyte counts (0 points when ≥ 1400/μL and 1 point when < 1400/μL) and κ/λ ratio (0 points when 0.1–10 and 1 point when < 0.1 or ≥ 10) before elotuzumab treatment (model 1). The median TTNT (months) and 95% CI are shown in the figure. The TTNT values were corrected by the prior regimen numbers and the prior use of daratumumab. NA indicates not applicable. (**B**) The overall survival (OS) of the MM patients treated with elotuzumab according to the scoring system of model 1: 0 points (*black*), 1 point (*red*) and 2 points (*blue*). Only the patients with the first use of elotuzumab were analyzed. The 2-year OS of each group and 95% CI are shown in the figure. The OS values were corrected by the prior regimen numbers and the prior use of daratumumab. (**C**) The TTNT of the MM patients treated with elotuzumab according to the scoring system of model 2: 0 points (*black*), 1 point (*red*) and 2 points (*blue*). Total scores were calculated according to the lymphocyte counts (0 points when ≥ 1400/μL and 1 point when < 1400/μL) and β_2_ microglobulin (B2MG; 0 points when 5.5 mg/L and 1 point when ≥ 5.5 mg/L) before elotuzumab treatment (model 2). The median TTNT (months) and 95% CI are shown in the figure. The TTNT values were corrected by the prior use of daratumumab. NA indicates not applicable. (**D**) The overall survival (OS) of the MM patients treated with elotuzumab according to the scoring system of model 2: 0 points (*black*), 1 point (*red*) and 2 points (*blue*). Only the patients with the 1^st^ use of elotuzumab were analyzed. The 2-year OS of each group and 95% CI are shown in the figure. The OS values were corrected by the prior use of daratumumab. The number of patients at risk in each group is shown in the lower panel of each figure. CI: confidence interval.

From these results, we proposed a new model to predict the durability of the effect (longer TTNT) of elotuzumab treatment. We first made a model using three factors: lymphocyte counts, κ/λ ratio and B2MG. Although we did not observe a strong correlation between the κ/λ ratio and B2MG, when we categorized the cases by all three factors, we found a strong correlation between the cases categorized by the κ/λ ratio and B2MG. Therefore, we divided the cases into two different multivariate models (model 1 and model 2) using one of these two factors (κ/λ ratio and B2MG) reflecting the tumor burden. We then classified the patients into three categories based on (1) the lymphocyte counts and κ/λ ratio (model 1) or (2) the lymphocyte counts and B2MG (model 2). We assigned 0 points to patients with lymphocyte counts of 1400/μl or more and 1 point to those with lymphocyte counts of less than 1400/μl. We also scored 0 points to patients having a κ/λ ratio of 0.1–10 and 1 point to those having a κ/λ ratio of less than 0.1 or 10 or more. We confirmed that the scoring system (model 1) was significantly correlated with the TTNT of elotuzumab treatment in multivariate analysis (Table 4). We also corrected the TTNT of our models with the prior regimen numbers and the prior use of daratumumab (Fig. 2A). The patients with a total score of 0 showed significantly longer TTNT compared to those with scores of 1 or 2 (*p* < 0.001, Fig. 2A). This result was confirmed by bootstrap methods (Table 4). Moreover, when we analyzed the OS after the elotuzumab treatment only for the patients with first elotuzumab treatment, we found that the patients with a total score of 0 or 1 showed significantly superior OS than the patients with a total score of 2 (*p* < 0.001, Fig. 2B). The c-index of model 1 was 0.728.

**Table 4:** Multivariate analysis for TTNT.

Factors		Model 1				Model 2			
		Hazard ratio	95% CI	*p*-value	*p*-value*	Hazard ratio	95% CI	*p*-value	*p*-value*
Type of treatment regimen	ERd	1		0.143	0.151	1		0.431	0.432
	EPd	0.713	0.453–1.121			0.801	0.460–1.393		
Lymphocyte Free light chain score	0	1		< 0.001	< 0.001				
	1	2.395	1.236–4.639						
	2	4.069	2.063–8.026						
Lymphocyte B2MG score	0					1		< 0.001	< 0.001
	1					2.123	1.178–3.829		
	2					4.608	2.144–9.904		
Prior regimen numbers	< 4	1		0.009	0.007	1		0.292	0.228
	≥ 4	1.967	1.178–3.285			1.358	0.768–2.399		
Prior use of daratumumab	No	1		0.021	0.005	1		< 0.001	< 0.001
	Yes	1.627	1.075–2.463			3.247	1.974–5.343		

Multivariate analyses against TTNT in MM patients treated with elotuzumab were performed using the scoring system (model 1 and model 2) as factors. In model 1, we picked up the following factors: type of treatment regimen, WBC counts, lymphocyte free light chain score, number of prior regimens and prior use of daratumumab. In model 2, we picked up the following factors: type of treatment regimen, WBC counts, lymphocyte B2MG score, number of prior regimens and prior use of daratumumab. The Cox proportional hazard model was used to calculate the hazard ratio for each variable; the 95% CI and *p*-value are shown. *P-value after the bootstrapping process (1000 samples).

TTNT, CI, ERd, EPd and B2MG. TTNT: time to next treatment; CI: confidence interval; ERd: elotuzumab, lenalidomide and dexamethasone regimen; EPd: elotuzumab, pomalidomide and dexamethasone regimen; B2MG: β_2_ microglobulin.

Next, we assigned the patients scores according to their lymphocyte counts in the same way as in model 1. We also scored the patients with a B2MG of less than 5.5 mg/L with 0 points and those with a B2MG of 5.5 mg/L or more with 1 point. The patients with a total score of 0 or 1 showed significantly longer TTNT compared to those with a score of 2 (*p* < 0.001, Fig. 2C). The scoring system (model 2) was significantly correlated with the TTNT of elotuzumab treatment in multivariate analysis, and this result was also confirmed by bootstrap methods (Table 4). The TTNT of model 2 was also corrected with prior use of daratumumab (Fig. 2C). When we analyzed the OS after elotuzumab treatment only for the patients with first elotuzumab treatment, we also found that the patients with total scores of 0 and 1 showed significantly superior OS compared to the patients with a total score of 2 (*p* < 0.001, Fig. 2D). The c-index for model 2 was 0.641. We applied these models to each treatment regimen and confirmed that both models fit both regimens (Figs. S8A,B and S9A,B).

Therefore, we conclude that by using this simple model, we could predict the patients who would exhibit a durable response (longer TTNT) to elotuzumab treatment (Fig. S10).

## Discussion

Repeated chemotherapy against cancer and hematological malignancy frequently leads to severe lymphopenia. Moreover, lymphopenia has long been associated with poor prognosis not only in lymphoma but also other cancers^13–15^. Recently, an association between the pretreatment lymphocyte count and the efficacy of immune checkpoint inhibitors was reported in patients with head and neck squamous cell carcinoma^25^. Also there is a report that the similarity of a patient's immune cell composition to that of healthy donors may have prognostic relevance at diagnosis and after ERd treatment in high risk smoldering myeloma^26^. We hypothesized that the efficacy of elotuzumab could be predicted by the balance between the tumor burden of myeloma and host immune cells. To prove our concept, we chose the κ/λ ratio and B2MG as candidate biomarkers for representing the tumor burden of myeloma. We also selected WBC and lymphocyte counts as indices of the host immune status. The present study demonstrated that the simple model using the κ/λ ratio (or B2MG), and lymphocyte counts easily predicted the durable efficacy of elotuzumab treatment. Patients with a total score of 0, who had a low tumor burden (non-deviated κ/λ ratio level or lower B2MG) and preserved host immune cells (higher lymphocyte counts), were the best candidates for elotuzumab treatment. They could obtain the benefit of elotuzumab treatment for long duration with better prognosis. Because a previous report showed that the neutrophil-to-lymphocyte ratio is correlated with the prognosis of MM^27^, we also analyzed neutrophil counts, monocyte counts, the neutrophil/lymphocyte ratio and the monocyte/lymphocyte ratio as indices of host immunity. However, none of these parameters was correlated with the TTNT of elotuzumab (data not shown). Our scoring system could identify the scenarios where elotuzumab might find itself more useful, the usefulness of our scoring system needs to be substantiated by other treatment for MM in other cohort studies.

Since soluble SLAMF7 (sSLAMF7) impaired anti-SLAMF7 antibody mediated ADCC activity, sSLAMF7 levels could be a predictive biomarker for elotuzumab therapy^28,29^. However, it is difficult to predict the best treatment response of elotuzumab beforehand at the present, since the measurement of sSLAMF7 is not available in clinical practice. Our study design focused on TTNT in order to establish a model for predicting patients with a durable response to elotuzumab treatment. When we compared the c-index values of each model, we found that the c-indices of model 1 and model 2 for the ERd regimen were 0.722 and 0.592, respectively. On the other hand, the c-indices of model 1 and model 2 for the EPd regimen were 0.693 and 0.76, respectively. We plan to use model 1 for future study because the c-index in model 1 was higher than that of model 2 as a whole, and also the κ/λ ratio is more frequently analyzed than B2MG in clinical practice in Japan.

We acknowledge that universal prognostic markers, such as B2MG or International Staging System (ISS), which mainly reflect tumor burden, correlated with the TTNT of elotuzumab treatment^2,6^. However, the c-index of our predictive models (model 1: 0.728, model 2: 0.641) are higher than that of B2MG (0.518) or of ISS (0.625). Also, by adding the parameters which correlate to host immune status to the universal prognostic markers, we could more efficiently select the proper patients for elotuzumab treatment as shown in score 1 in model 1 and model 2. Our results indicate that elotuzumab treatments were particularly effective to those with higher lymphocyte counts. These results are supported by the previous reports which demonstrated that elotuzumab could change the immune-suppressive tumor microenvironment to enhance anti-tumor effect by blocking SLAMF7 signaling and eliminating immunosuppressive T cells^30–32^. Although the TTNT of the patients with a total score of 1 was shorter than the TTNT of the patients with a total score of 0 in model 1, the OS after the first elotuzumab treatment was nearly equivalent to that of the patients with a total score of 0. The same trend was also observed in model 2. Although the durability of elotuzumab treatment for patients with a total score of 1 was limited, we consider that the following treatment after elotuzumab improved the prognosis of these patients. It is also worth mentioning that the elotuzumab treatment did not interfere with the subsequent treatment regimens in these patients. Therefore, elotuzumab is a suitable treatment option for both types of patients—i.e., those with a total score of 0 or 1. Because the patients with a score of 2 showed shorter TTNT of elotuzumab treatment and worse prognosis after the first elotuzumab treatment compared to those with a score of 0 or 1, we might consider a treatment other than elotuzumab for these patients.

The present study also showed that the effectiveness of elotuzumab was attenuated by the prior use of daratumumab. The mechanism of action of elotuzumab in MM patients involves the activation of natural killer (NK) cells through both CD16-mediated antibody dependent cellular cytotoxicity and direct co-stimulation via engagement with SLAMF7 as well as promoting antibody-dependent cellular phagocytosis by macrophages^7,8,33^. As it has been reported that daratumumab decreases the number of NK cells, the depletion of NK cells via daratumumab could attenuate the effectiveness of elotuzumab^7,8,33,34^. Since we do not routinely check the NK cell counts in practice, we could not confirm the existence of this mechanism. However, higher lymphocyte counts may be a prerequisite for the higher NK cell counts, which needs to be confirmed by another study. We interpreted that the sequence of treatment is important. As daratumumab is more frequently used as a front- or early-line therapy today, candidates for elotuzumab treatment are limited to patients with a low tumor burden after treatment with protease inhibitors and/or immunomodulatory drugs without daratumumab treatment. As the effectiveness of elotuzumab could be attenuated by the prior use of daratumumab, it might be useful to use daratumumab after elotuzumab treatment.

There are limitations in this study. First, this was a retrospective observation study in which the individual physicians decided the treatment. Because this was not a randomized prospective study, a potential selection bias for elotuzumab treatment cannot be ruled out and could not be controlled for in the multivariate analysis. We adapted boot strap method to perform internal validation. However, it was difficult to confirm external validation in our cohort because of the limited number of analyzed patients. Therefore, prospective cohort studies of elotuzumab treatment will be needed to substantiate our results. Second, we could not include the data regarding high-risk cytogenetic abnormalities in our multivariate analysis due to limited data. As high-risk cytogenic abnormalities are one of important prognostic factors, this should also be validated by other studies. Third, we could not analyze the detailed fraction of lymphocytes (such as CD4 + T cells, CD8 + T cells, regulatory T cell, etc.) for further understating the mechanism of elotuzumab. In spite of these limitations, to our knowledge this is the first study to demonstrate that the efficacy of elotuzumab, and potentially other immunotherapies could be predicted by the balance between the tumor burden and host immune status. The strength of our study lies in the point that we tried to identify the predictive markers for elotuzumab treatment using the factors which are easily available in actual clinical practice.

In conclusion, we proposed a new scoring system using lymphocyte counts and the κ/λ ratio or B2MG to predict the TTNT of elotuzumab treatment. This scoring system would be useful for differentiating patients who could benefit from elotuzumab treatment.

## Supplementary Information

Supplementary Figures.

## Data Availability

The KMF database is only available to the KMF members. However, the data of this study are available from the corresponding author, JK, upon reasonable request.
