# Supplementary material for: Efficacy of elotuzumab for multiple myeloma in reference to lymphocyte counts and kappa/lambda ratio or B2 microglobulin
Source: Sci Rep. 2023 Mar 29;13:5159. doi: 10.1038/s41598-023-32426-6 (PMC10060246; doi:10.1038/s41598-023-32426-6)
Supplement: Supplementary file 1 — Supplementary Figures. [file 41598_2023_32426_MOESM1_ESM.pdf]

Supplemental Materials

Efficacy of elotuzumab for multiple myeloma in reference to lymphocyte counts and kappa/lambda ratio or B2 microglobulin

Yutaka Shimazu[1], Junya Kanda[1*], Satoru Kosugi[2], Tomoki Ito[3], Hitomi Kaneko[4], Kazunori Imada[4], Yuji Shimura[5], Shin-ichi Fuchida[6], Kentaro Fukushima[7], Hirokazu Tanaka[8], Satoshi Yoshihara[9], Kensuke Ohta[10], Nobuhiko Uoshima[11], Hideo Yagi[12], Hirohiko Shibayama[13], Ryosuke Yamamura[14], Yasuhiro Tanaka[15], Hitoji Uchiyama[16], Yoshiyuki Onda[17], Yoko Adachi[18], Hitoshi Hanamoto[19], Ryoichi Takahashi[20], Mitsuhiro Matsuda[21], Takashi Miyoshi[22], Teruhito Takakuwa[23], Masayuki Hino[23], Naoki Hosen[7], Shosaku Nomura[3], Chihiro Shimazaki[6], Itaru Matsumura[8], Akifumi Takaori-Kondo[1] and Junya Kuroda[5], Kansai Myeloma Forum[24]

Inventory of Supplemental Information

Supplemental Figure Legends

Supplemental Figures

Figure S1.

Figure S2.

Figure S3.

Figure S4.

Figure S5.

Figure S6.

Figure S7.

Figure S8.

Figure S9.

Figure S10.

Supplemental Figure Legends

Figure S1. (A-F) The histogram of white blood cell counts **(A)**, neutrophil counts **(B)**, monocyte counts **(C)**, lymphocyte counts **(D)**, β_2 microglobulin (B2MG) **(D)** and κ/λ ratio **(E)**. The horizontal axis was plot in log scale. The median, 25th percentile and 75th percentile values are described in the left upper panel of each figure.

Figure S2. (A) The time to next treatment (TTNT) of the multiple myeloma (MM) patients according to the β_2 microglobulin (B2MG) level: B2MG less than 3.5 mg/L (*black*), 3.5 mg/L or more to less than 5.5 mg/L (*red*) and 5.5 mg/L or more (*red*). The median TTNT (months) and 95% CI are shown. The hazard ratio (HR) of B2MG <3.5mg/L as a reference and 95% CI are also described. **(B)** TTNT of the MM patients according to the white blood cell (WBC) counts: less than 2500/μL (*black*), 2500/μL or more to less than 3500/μL (*red*), 3500/μL or more to less than 4500/μL (*blue*) and 4500/μL or more (*green*). The median TTNT (months) and 95% CI are shown. The HR of WBC counts <2500/μL and 95% CI are also described as a reference. **(C)** TTNT of the MM patients according to the lymphocyte counts: less than 700/μL (*black*), 700/μL or more to less than 1000/μL (*red*), 1000/μL or more to less than 1400/μL (*blue*) and 1400/μL or more (*green*). The median TTNT (months) and 95% CI are shown. The HR of lymphocyte counts <700/μL and 95% CI are also described as a reference. **(D)** TTNT of the MM patients according to the neutrophile counts: less than 1000/μL (*black*), 1000/μL or more to less than 2000/μL (*red*), 2000/μL or more to less than 3000/μL (*blue*) and 3000/μL or more (*green*). The median TTNT (months) and 95% CI are shown. The HR of neutrophile counts <1000/μL and 95% CI are also described as a reference. **(E)** TTNT of the MM patients according to the monocyte counts: less than 200/μL (*black*), 200/μL or

more to less than 300/μL (*red*), 300/μL or more to less than 400/μL (*blue*) and 400/μL or more (*green*). The median TTNT (months) and 95% CI are shown. The HR of monocyte counts <200/μL and 95% CI are also described as a reference. The number of patients at risk in each group is shown in the lower panel of each figure.

Abbreviations: CI. CI: confidence interval.

Figure S3. The proportion of the best treatment response against elotuzumab treatment. The overall response rate includes CR, VGPR and PR.

Abbreviations: CR, VGPR, PR, SD and PD. CR: complete remission; VGPR: very good partial response; PR: partial response; SD: stable disease; PD: progressive disease.

Figure S4. (A) The time to next treatment (TTNT) of the multiple myeloma (MM) patients according to the treatment regimen: elotuzumab, lenalidomide and dexamethasone (ERd, *black*) and elotuzumab, pomalidomide and dexamethasone (EPd, *red*). The median TTNT (months) and 95% CI are shown. **(B)** The TTNT of the MM patients according to the number of elotuzumab treatments: first time (*black*) and second time (*red*). The median TTNT (months) and 95% CI are shown. The number of patients at risk in each group is shown in the lower panel of each figure.

Abbreviations: CI. CI: confidence interval.

Figure S5. (A) The TTNT of the MM patients treated with elotuzumab according to the neutrophil counts: less than 3000/μL (*black*) and 3000/μL or more (*red*). **(B)** The TTNT of the MM patients treated with elotuzumab according to the monocyte counts: less than 300/μL (*black*) and 300/μL or more (*red*). The number of patients at risk in each group is

shown in the lower panel of each figure.

Abbreviations: CI. CI: confidence interval.

Figure S6. (A) The TTNT of the MM patients treated with elotuzumab according to the prior regimen number: fewer than 4 prior regimens (*black*) and 4 or more prior regimens (*red*). The median TTNT (months) and 95% CI are shown. NA indicates not applicable. **(B)** The TTNT of the MM patients treated with elotuzumab according to prior daratumumab use: nonuse of daratumumab (*black*) and prior use of daratumumab (*red*). The median TTNT (months) and 95% CI are shown. **(C)** The TTNT of the MM patients treated with elotuzumab according to the period from prior daratumumab use: less than 6 months (*black*) and 6 months or more (*red*). The median TTNT (months) and 95% CI are shown. The number of patients at risk in each group is shown in the lower panel of each figure.

Abbreviations: CI. CI: confidence interval.

Figure S7. Comparing the lymphocyte counts between prior use of daratumumab (not used or previously used). Average lymphocyte counts with standard deviation (SD) of each group were shown in the figure.

Figure S8. (A) The TTNT of the MM patients treated with elotuzumab, lenalidomide and dexamethasone (ERd) regimen according to the scoring system used in model 1: 0 points (*black*), 1 point (*red*) and 2 points (*blue*). Total scores were calculated according to the lymphocyte counts (0 points when ≥1400/μL and 1 point when <1400/μL) and κ/λ ratio (0 points when 0.1-10 and 1 point when <0.1 or ≥10) before elotuzumab treatment

(model 1). The median TTNT (months) and 95% CI values are shown in the figure. The hazard ratio (HR) of total score 0 as a reference with 95% CI is also described. The TTNT values were corrected by the following factors: prior treatment regimen and the use of daratumumab before. **(B)** The TTNT of the MM patients treated with a regimen of elotuzumab, pomalidomide and dexamethasone (EPd) according to the scoring system used in model 1 and described above in panel **(A)**. The median TTNT (months) and 95% CI are shown in the figure. The hazard ratio (HR) of total score 0 as a reference with 95% CI is also described. The TTNT values were corrected by the following factors: prior treatment regimen and prior use of daratumumab. The number of patients at risk in each group is shown in the lower panel of each figure.

Abbreviations: CI and NA. CI: confidence interval; NA: not applicable.

Figure S9. (A) The TTNT of the MM patients treated with a regimen of elotuzumab, pomalidomide and dexamethasone (EPd) according to the scoring system used in model 1: 0 points (*black*), 1 point (*red*) and 2 points (*blue*). Total scores were calculated according to the lymphocyte counts (0 points when ≥1400/µL and 1 point when <1400/µL) and B2MG (0 points when less than 5.5mg/L and 1 point when 5.5mg/L or more) before elotuzumab treatment (model 2). The median TTNT (months) and 95% CI are shown in the figure. The hazard ratio (HR) of the total score 0 and 95% CI are also described as a reference. The TTNT values were corrected by the prior use of daratumumab. **(B)** The TTNT of the MM patients treated with a regimen of elotuzumab, pomalidomide and dexamethasone (EPd) according to the scoring system (model 1) as in panel **(A)**. The median TTNT (months) and 95% CI are shown in the figure. The hazard ratio (HR) of the total score 0 and 95% CI are also described as a reference. The TTNT

were corrected by the prior use of daratumumab. The number of patients at risk in each group is shown in the lower panel of each figure.

Abbreviations: CI and NA. CI: confidence interval; NA: not applicable.

Figure S10. The treatment algorithm according to the scoring system.

Figure S1

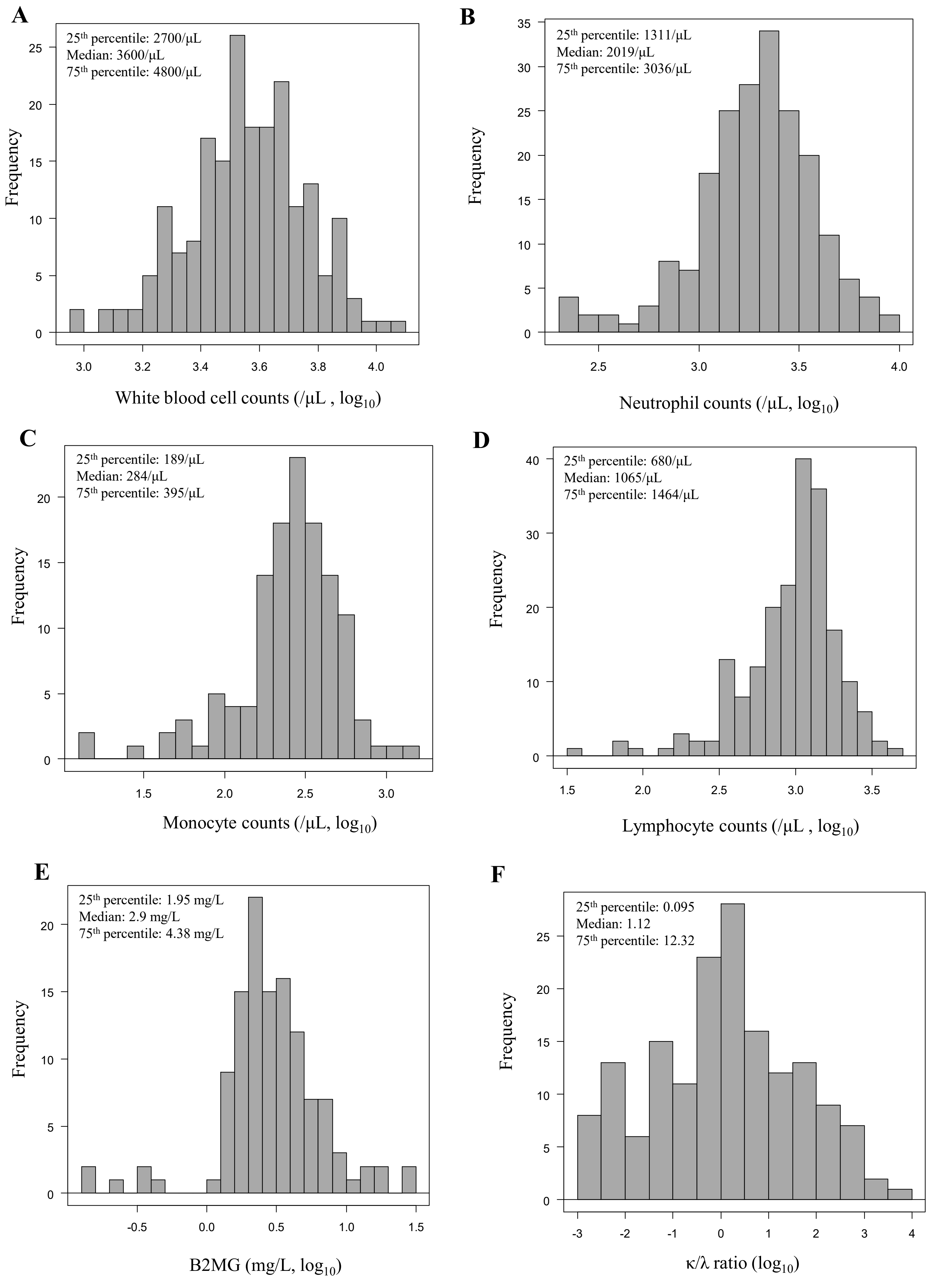
A
25th percentile: 2700/µL
Median: 3600/µL
75th percentile: 4800/µL
Frequency
White blood cell counts (/µL , log10)
B
25th percentile: 1311/µL
Median: 2019/µL
75th percentile: 3036/µL
Frequency
Neutrophil counts (/µL, log10)
C
25th percentile: 189/µL
Median: 284/µL
75th percentile: 395/µL
Frequency
Monocyte counts (/µL, log10)
D
25th percentile: 680/µL
Median: 1065/µL
75th percentile: 1464/µL
Frequency
Lymphocyte counts (/µL , log10)
E
25th percentile: 1.95 mg/L
Median: 2.9 mg/L
75th percentile: 4.38 mg/L
Frequency
B2MG (mg/L, log10)
F
25th percentile: 0.095
Median: 1.12
75th percentile: 12.32
Frequency
κ/λ ratio (log10)

Figure S2

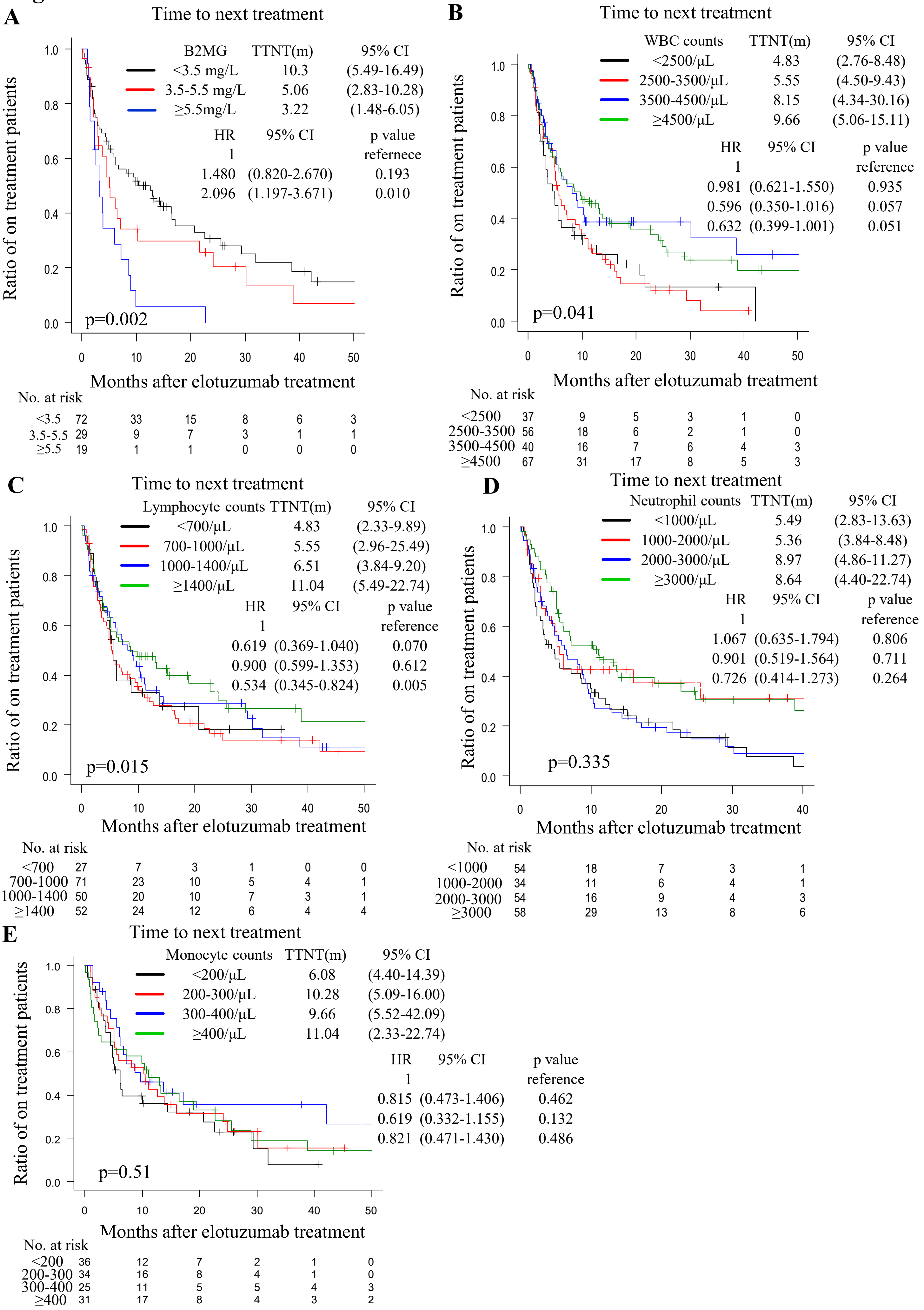

A
Time to next treatment
B2MG TTNT(m) 95% CI
<3.5 mg/L 10.3 (5.49-16.49)
3.5-5.5 mg/L 5.06 (2.83-10.28)
≥5.5mg/L 3.22 (1.48-6.05)
HR 95% CI p value
1 refernece
1.480 (0.820-2.670) 0.193
2.096 (1.197-3.671) 0.010
p=0.002
Ratio of on treatment patients
Months after elotuzumab treatment
No. at risk
<3.5 72 33 15 8 6 3
3.5-5.5 29 9 7 3 1 1
≥5.5 19 1 1 0 0 0
B
Time to next treatment
WBC counts TTNT(m) 95% CI
<2500/μL 4.83 (2.76-8.48)
2500-3500/μL 5.55 (4.50-9.43)
3500-4500/μL 8.15 (4.34-30.16)
≥4500/μL 9.66 (5.06-15.11)
HR 95% CI p value
1 reference
0.981 (0.621-1.550) 0.935
0.596 (0.350-1.016) 0.057
0.632 (0.399-1.001) 0.051
p=0.041
Ratio of on treatment patients
Months after elotuzumab treatment
No. at risk
<2500 37 9 5 3 1 0
2500-3500 56 18 6 2 1 0
3500-4500 40 16 7 6 4 3
≥4500 67 31 17 8 5 3
C
Time to next treatment
Lymphocyte counts TTNT(m) 95% CI
<700/μL 4.83 (2.33-9.89)
700-1000/μL 5.55 (2.96-25.49)
1000-1400/μL 6.51 (3.84-9.20)
≥1400/μL 11.04 (5.49-22.74)
HR 95% CI p value
1 reference
0.619 (0.369-1.040) 0.070
0.900 (0.599-1.353) 0.612
0.534 (0.345-0.824) 0.005
p=0.015
Ratio of on treatment patients
Months after elotuzumab treatment
No. at risk
<700 27 7 3 1 0 0
700-1000 71 23 10 5 4 1
1000-1400 50 20 10 7 3 1
≥1400 52 24 12 6 4 4
D
Time to next treatment
Neutrophil counts TTNT(m) 95% CI
<1000/μL 5.49 (2.83-13.63)
1000-2000/μL 5.36 (3.84-8.48)
2000-3000/μL 8.97 (4.86-11.27)
≥3000/μL 8.64 (4.40-22.74)
HR 95% CI p value
1 reference
1.067 (0.635-1.794) 0.806
0.901 (0.519-1.564) 0.711
0.726 (0.414-1.273) 0.264
p=0.335
Ratio of on treatment patients
Months after elotuzumab treatment
No. at risk
<1000 54 18 7 3 1
1000-2000 34 11 6 4 1
2000-3000 54 16 9 4 3
≥3000 58 29 13 8 6
E
Time to next treatment
Monocyte counts TTNT(m) 95% CI
<200/μL 6.08 (4.40-14.39)
200-300/μL 10.28 (5.09-16.00)
300-400/μL 9.66 (5.52-42.09)
≥400/μL 11.04 (2.33-22.74)
HR 95% CI p value
1 reference
0.815 (0.473-1.406) 0.462
0.619 (0.332-1.155) 0.132
0.821 (0.471-1.430) 0.486
p=0.51
Ratio of on treatment patients
Months after elotuzumab treatment
No. at risk
<200 36 12 7 2 1 0
200-300 34 16 8 4 1 0
300-400 25 11 5 5 4 3
≥400 31 17 8 4 3 2

Figure S3

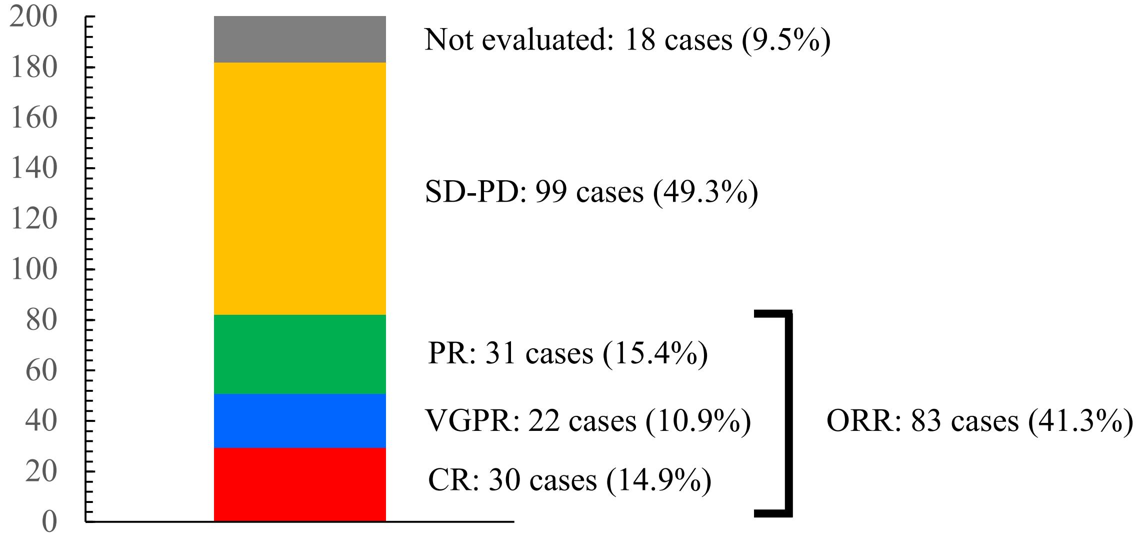
200
180
160
140
120
100
80
60
40
20
0
Not evaluated: 18 cases (9.5%)
SD-PD: 99 cases (49.3%)
PR: 31 cases (15.4%)
VGPR: 22 cases (10.9%)
CR: 30 cases (14.9%)
ORR: 83 cases (41.3%)

Figure S4

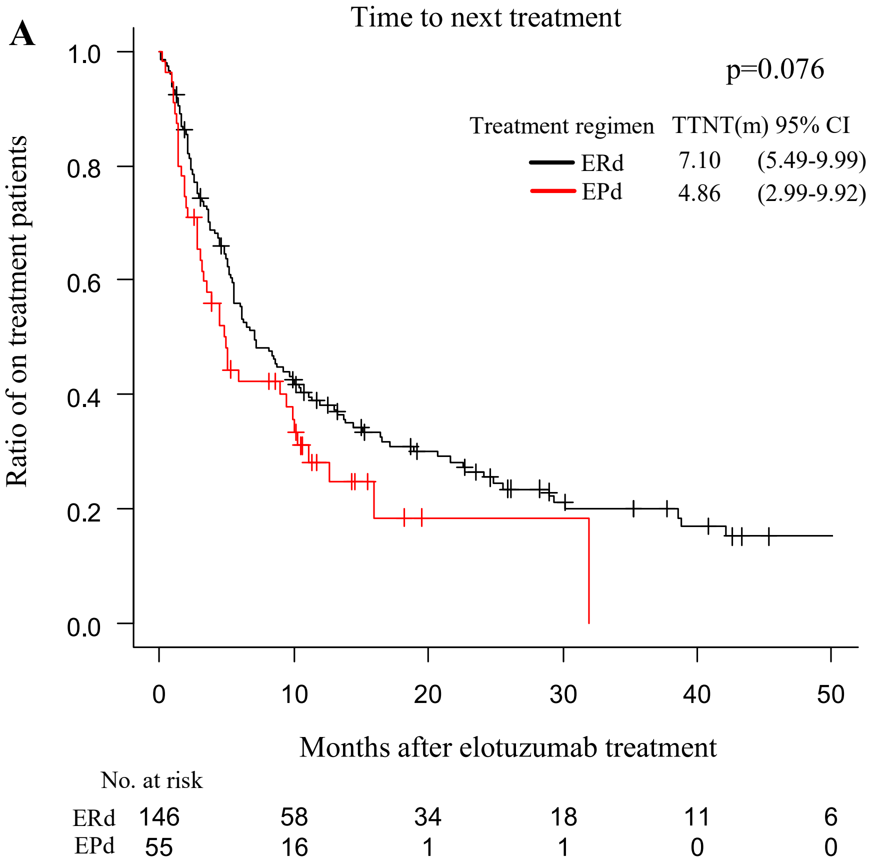
A
Time to next treatment
p=0.076
Treatment regimen TTNT(m) 95% CI
ERd 7.10 (5.49-9.99)
EPd 4.86 (2.99-9.92)
Ratio of on treatment patients
1.0
0.8
0.6
0.4
0.2
0.0
0 10 20 30 40 50
Months after elotuzumab treatment
No. at risk
ERd 146 58 34 18 11 6
EPd 55 16 1 1 0 0

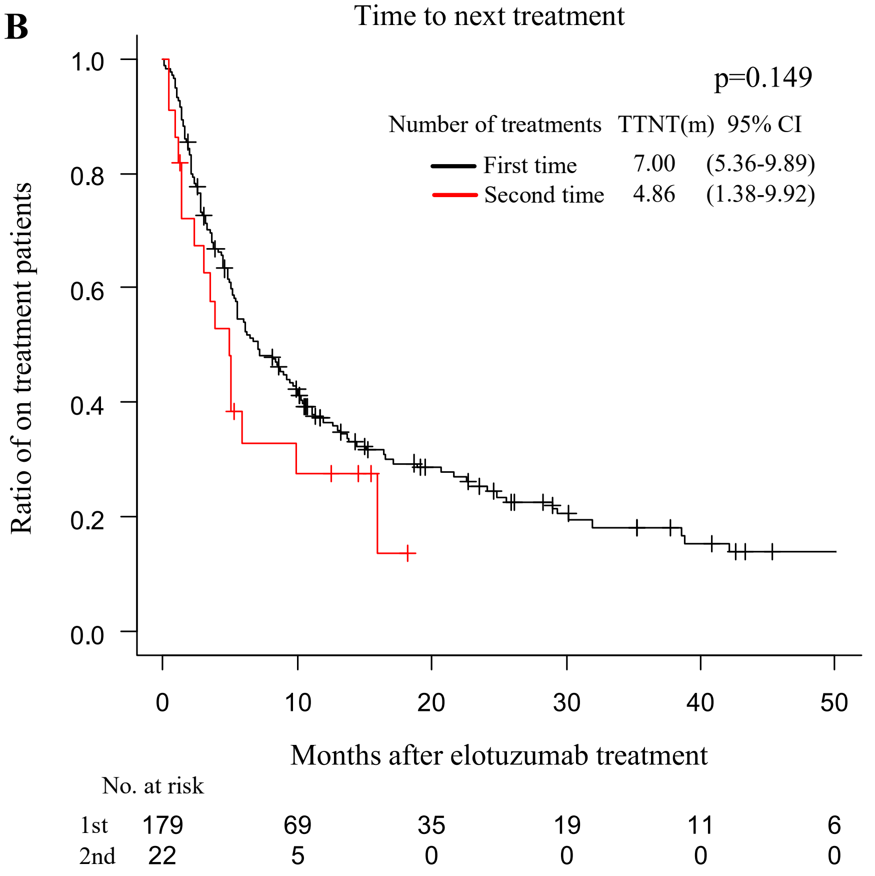
B
Time to next treatment
p=0.149
Number of treatments TTNT(m) 95% CI
First time 7.00 (5.36-9.89)
Second time 4.86 (1.38-9.92)
Ratio of on treatment patients
1.0
0.8
0.6
0.4
0.2
0.0
0 10 20 30 40 50
Months after elotuzumab treatment
No. at risk
1st 179 69 35 19 11 6
2nd 22 5 0 0 0 0

Figure S5

A

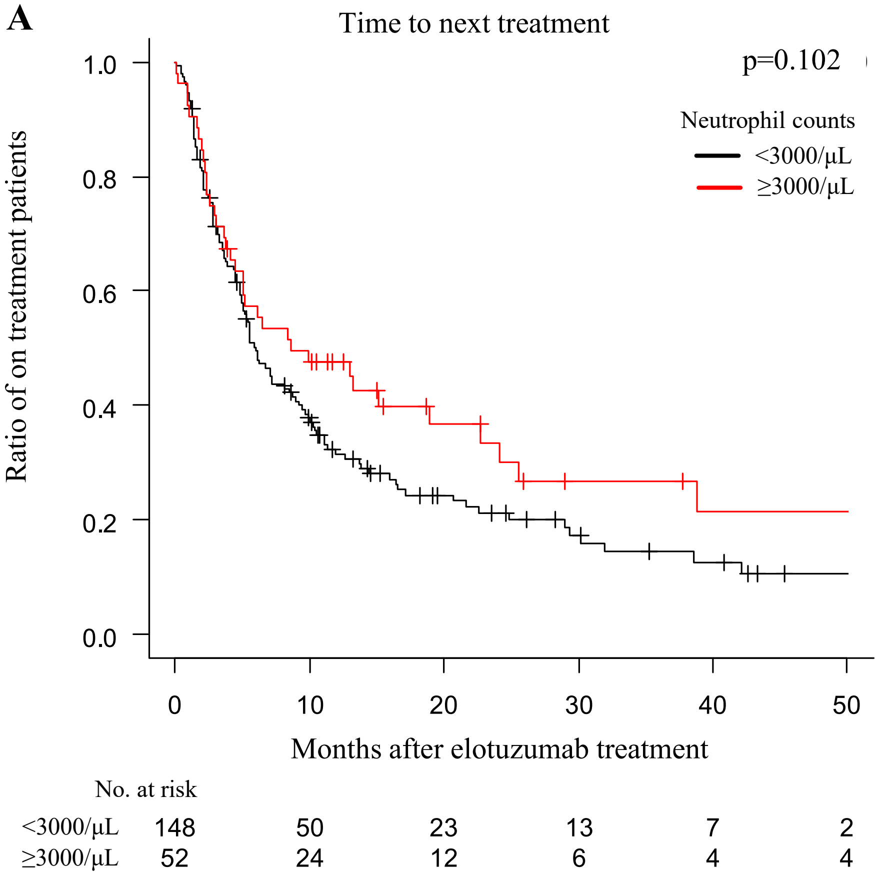

Time to next treatment
p=0.102
Neutrophil counts
<3000/μL
≥3000/μL
Ratio of on treatment patients
1.0
0.8
0.6
0.4
0.2
0.0
0
10
20
30
40
50
Months after elotuzumab treatment
No. at risk
<3000/μL 148 50 23 13 7 2
≥3000/μL 52 24 12 6 4 4

B

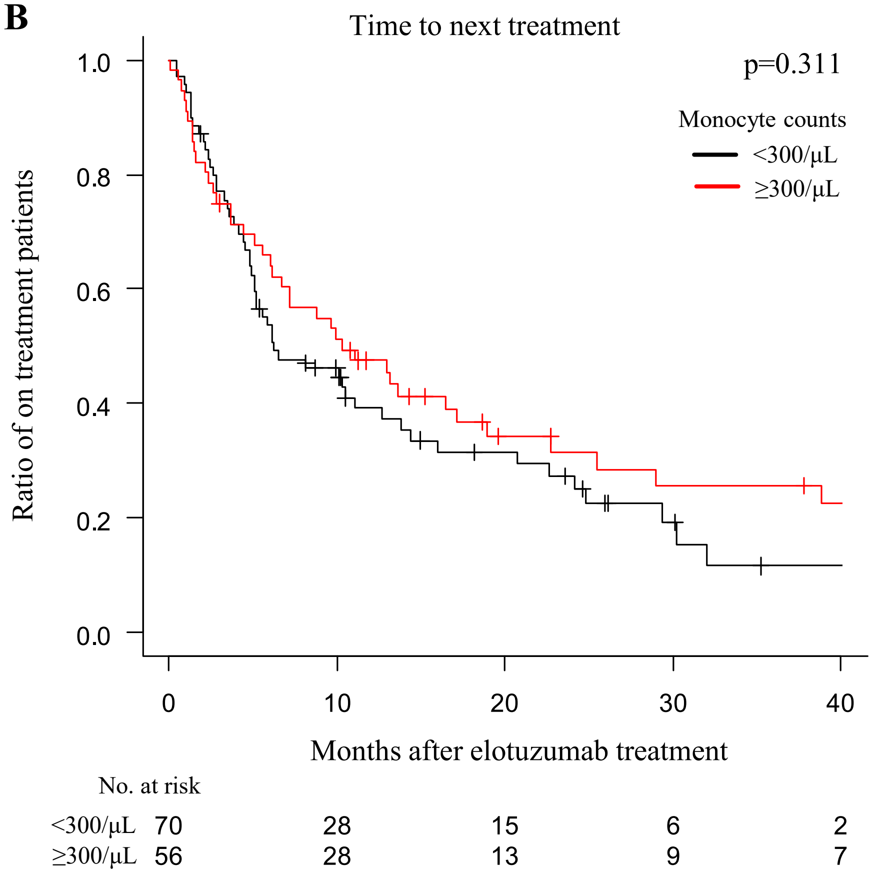

Time to next treatment
p=0.311
Monocyte counts
<300/μL
≥300/μL
Ratio of on treatment patients
1.0
0.8
0.6
0.4
0.2
0.0
0
10
20
30
40
Months after elotuzumab treatment
No. at risk
<300/μL 70 28 15 6 2
≥300/μL 56 28 13 9 7

Figure S6

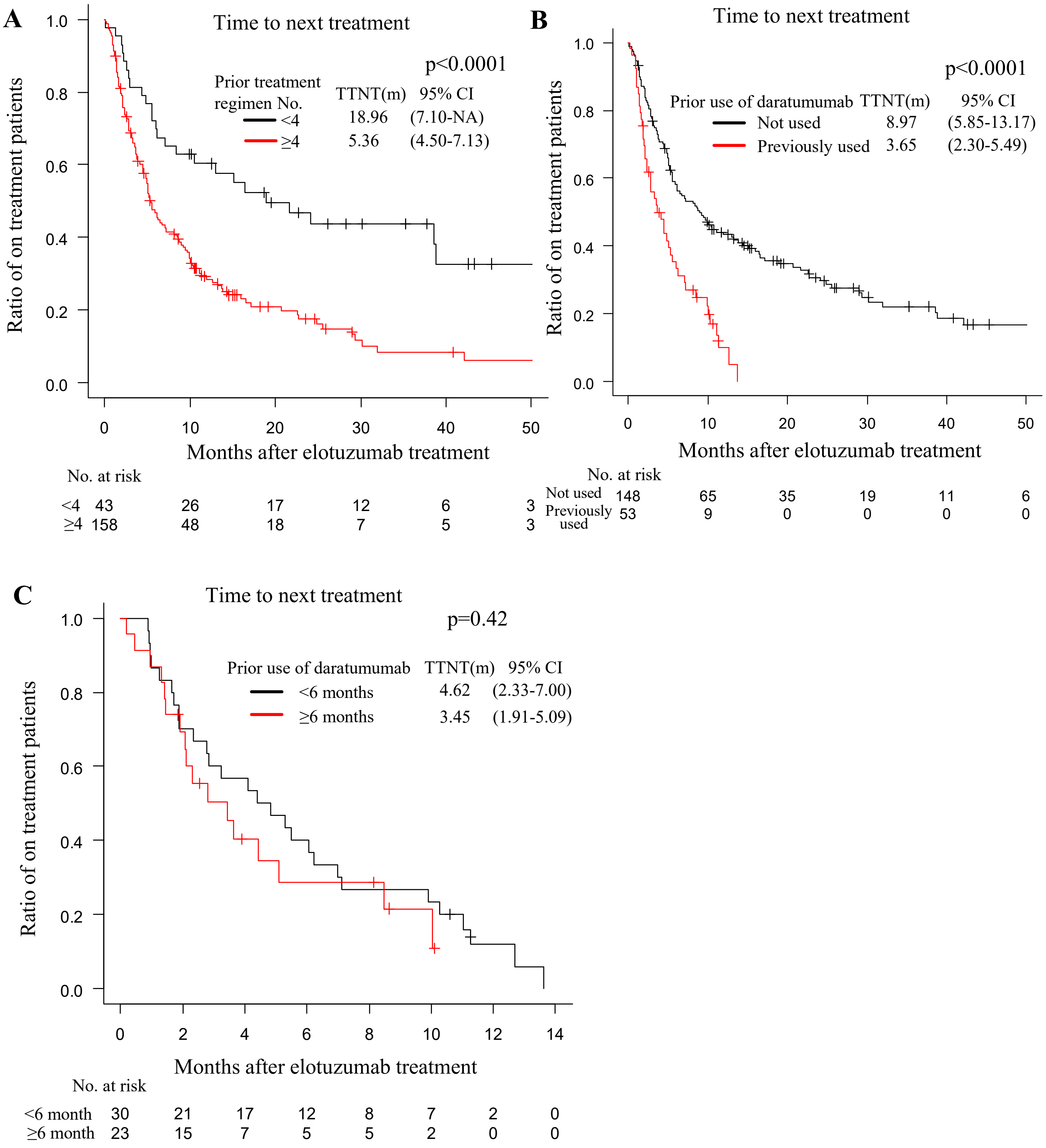
A
Time to next treatment
p<0.0001
Prior treatment regimen No.
TTNT(m) 95% CI
<4 18.96 (7.10-NA)
≥4 5.36 (4.50-7.13)
Ratio of on treatment patients
Months after elotuzumab treatment
No. at risk
<4 43 26 17 12 6 3
≥4 158 48 18 7 5 3
B
Time to next treatment
p<0.0001
Prior use of daratumumab TTNT(m) 95% CI
Not used 8.97 (5.85-13.17)
Previously used 3.65 (2.30-5.49)
Ratio of on treatment patients
Months after elotuzumab treatment
No. at risk
Not used 148 65 35 19 11 6
Previously used 53 9 0 0 0 0
C
Time to next treatment
p=0.42
Prior use of daratumumab TTNT(m) 95% CI
<6 months 4.62 (2.33-7.00)
≥6 months 3.45 (1.91-5.09)
Ratio of on treatment patients
Months after elotuzumab treatment
No. at risk
<6 month 30 21 17 12 8 7 2 0
≥6 month 23 15 7 5 5 2 0 0

Figure S7

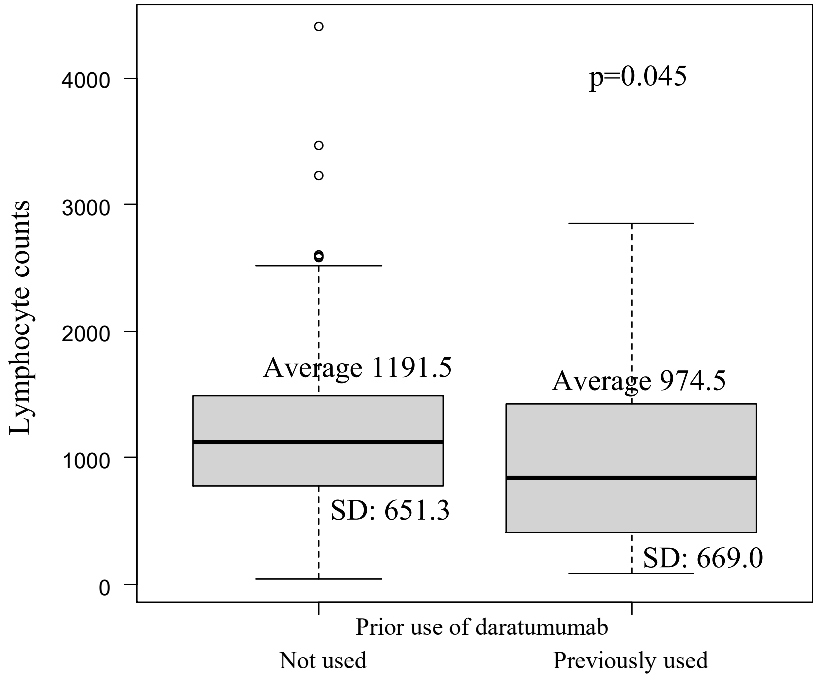
p=0.045
Lymphocyte counts
4000
3000
2000
1000
0
Average 1191.5
Average 974.5
SD: 651.3
SD: 669.0
Prior use of daratumumab
Not used
Previously used

Figure S8

A

Time to next treatment

ERd regimen

p<0.001

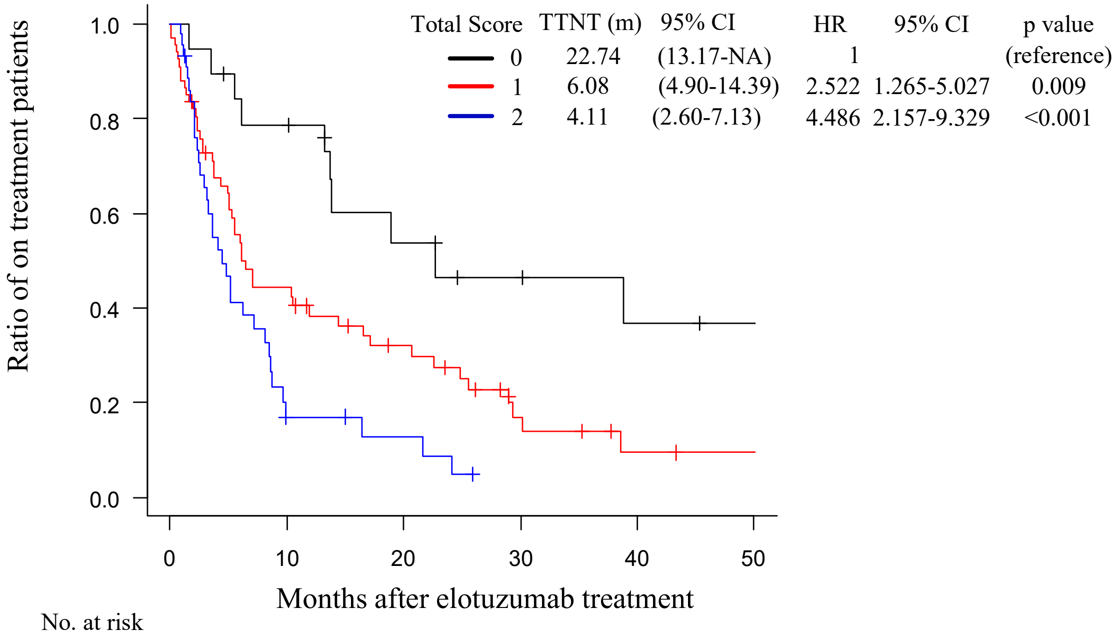

No. at risk

0	20	15	9	6	4	3
1	62	26	16	6	2	1
2	38	5	3	0	0	0

Score	Lymphocyte counts	κ/λ ratio
0	≥1400	0.1-10
1	<1400	<0.1, ≥10

B

Time to next treatment

EPd regimen

p=0.028

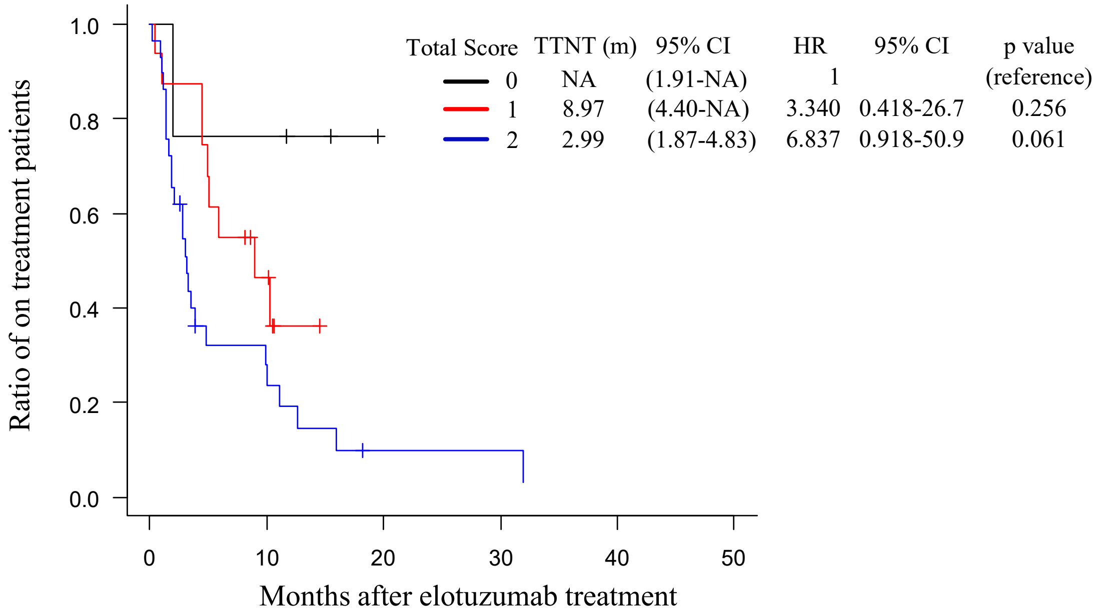

No. at risk

0	4	3	0	0	0	0
1	15	5	0	0	0	0
2	28	6	1	1	0	0

Score	Lymphocyte counts	κ/λ ratio
0	≥1400	0.1-10
1	<1400	<0.1, ≥10

Figure S9

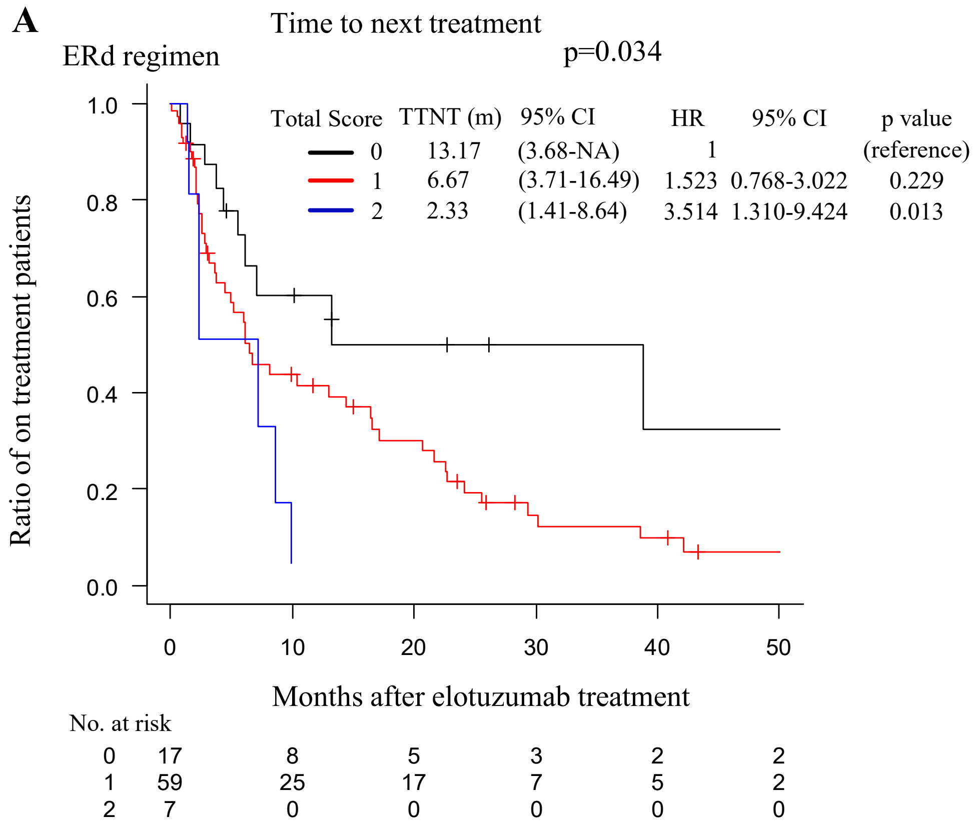

Score	Lymphocyte counts	B2MG
0	≥1400	<5.5mg/L
1	<1400	≥5.5mg/L

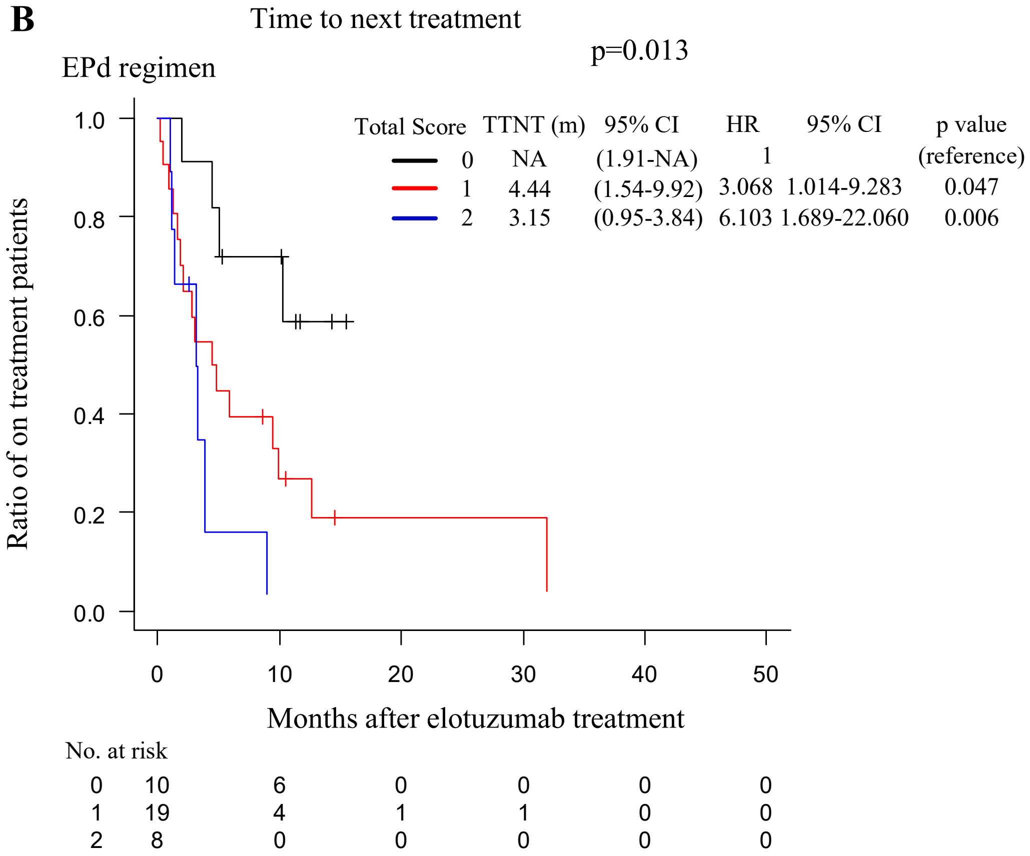

Score	Lymphocyte counts	B2MG
0	≥1400	< 5.5mg/L
1	<1400	≥5.5mg/L

Figure S10

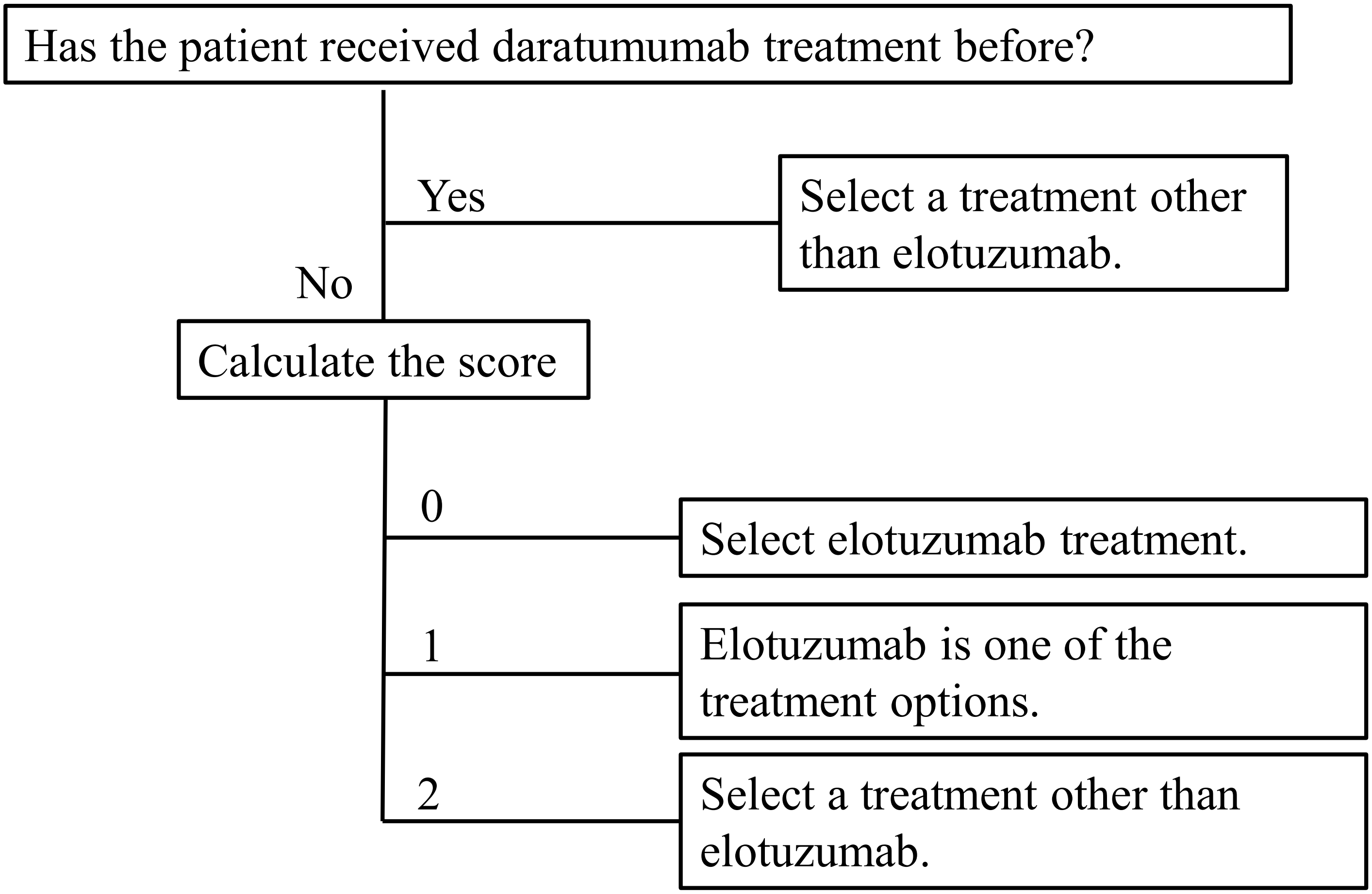
Has the patient received daratumumab treatment before?
Yes
Select a treatment other than elotuzumab.
No
Calculate the score
0
Select elotuzumab treatment.
1
Elotuzumab is one of the treatment options.
2
Select a treatment other than elotuzumab.
